# Measuring Tree Properties and Responses Using Low-Cost Accelerometers

**DOI:** 10.3390/s17051098

**Published:** 2017-05-11

**Authors:** Tim van Emmerik, Susan Steele-Dunne, Rolf Hut, Pierre Gentine, Marceau Guerin, Rafael S. Oliveira, Jim Wagner, John Selker, Nick van de Giesen

**Affiliations:** 1 Water Resources Section, Delft University of Technology, Delft 2628 CN, The Netherlands; s.c.steele-dunne@tudelft.nl (S.S.-D.); r.w.hut@tudelft.nl (R.H.); n.c.vandegiesen@tudelft.nl (N.v.d.G.); 2 Department of Earth and Environmental Engineering, Columbia University, New York, NY 10027, USA; pg2328@columbia.edu (P.G.); mg3237@columbia.edu (M.G.); 3 Department of Plant Biology, Institute of Biology, University of Campinas, Campinas, SP 13083-862, Brazil; rafaelsoliv@gmail.com; 4 Oregon Research Electronics, Tangent, OR 97389, USA; wagnejam99@comcast.net; 5 Department of Biological and Ecological Engineering, Oregon State University, Corvallis, OR 97331, USA; john.selker@oregonstate.edu

**Keywords:** hydrology, tree physiology, tree sway, wind, canopy, Amazon, drag coefficient, climate change, turbulence, interception

## Abstract

Trees play a crucial role in the water, carbon and nitrogen cycle on local, regional and global scales. Understanding the exchange of momentum, heat, water, and CO${}_{2}$ between trees and the atmosphere is important to assess the impact of drought, deforestation and climate change. Unfortunately, ground measurements of tree properties such as mass and canopy interception of precipitation are often expensive or difficult due to challenging environments. This paper aims to demonstrate the concept of using robust and affordable accelerometers to measure tree properties and responses. Tree sway is dependent on mass, canopy structure, drag coefficient, and wind forcing. By measuring tree acceleration, we can relate the tree motion to external forcing (e.g., wind, precipitation and related canopy interception) and tree physical properties (e.g., mass, elasticity). Using five months of acceleration data of 19 trees in the Brazilian Amazon, we show that the frequency spectrum of tree sway is related to mass, canopy interception of precipitation, and canopy–atmosphere turbulent exchange.

## 1. Introduction

Trees are important contributors to the local, regional, and global water and carbon cycle [1,2,3]. Trees play a key role in the carbon cycle as they store carbon as a result of their primary production. Through photosynthesis, carbon is assimilated for biomass production, and oxygen is released into the atmosphere. During this process, water is transpired through the stomata for cooling and is redistributed throughout the plant. Transpiration by trees accounts for most of the total evaporation from land on the global scale, making them a dominant contributor to the global water cycle [2,4]. The role of trees in the water cycle is even higher in tropical rainforests, where transpiration makes up to 70% of total evaporation [2]. Through transpiration, trees also have a large influence on soil moisture and groundwater, as their roots take up water to transport it to the stomata. Finally, canopy interception of precipitation makes up a significant part of the water cycle, as it can amount to up to 15–50% of precipitation [5,6].

The exchange of CO${}_{2}$ and water are determined by the turbulent exchange between the canopy and the atmosphere [7,8]. Vegetation is a rough surface that interacts with the atmospheric boundary layer [9]. Higher turbulence results in higher rates of CO${}_{2}$ and water exchange through increased mixing [10]. Studying the momentum transfer from wind to trees through tree sway might therefore yield information on turbulent regulation of tree photosynthesis and transpiration rates.

Measurements of tree properties, such as mass, canopy interception, and tree–atmosphere interaction, offer additional insights in the role of trees in the water and carbon cycle, and how this might be affected by drought, climate change, and deforestation. Some changes in drought-induced tree mortality and tree species distribution in response to climate change have been observed [11,12]. In addition, it has been shown that tropical deforestation results in warmer, drier conditions at local scales [13]. However, recent analyses are not conclusive, and various studies have highlighted the lack of broad empirical assessments [14]. Recent developments in remote sensing have allowed new analyses of the effects of droughts on (tropical) forests. As shown in previous work on forests and crops [15,16,17], diurnal differences in vegetation water content can be associated with water stress, which could have a significant effect on radar backscatter. Additional ground measurements are necessary to relate tree properties to spaceborne radar backscatter. Other work by Huete et al. [18] found a green-up of the vegetation in the Amazon during severe droughts using Moderate-resolution Imaging Spectroradiometer (MODIS) spectral imagery, which led to a long discussion about the possible explanations for these observations. Morton et al. [19] recently hypothesized that the original analyses were in fact based on an optical illusion, and that no actual green-up of the forest occurred. This exposed the critical lack of ground-based observations of tree properties for calibration and validation of remote sensing data [20].

Unfortunately, key variables and fluxes such as transpiration, mass, interception capacity, canopy drag, and turbulent exchange are not easy to measure accurately. Conventional measurement equipment, such as sap flow sensors and dendrometers, use invasive techniques to measure tree water fluxes [21] or are often expensive. Furthermore, these devices are not always robust enough to withstand the challenging field conditions in environments such as tropical forests. Using dendrometers that only measure stem diameter might also give inaccurate results, as it is required to know the relative contributions of the phloem and bark [22]. Few methods exist to measure canopy interception and throughfall, and they often involve complicated and labor intensive techniques [23]. Finally, many tree measurement techniques are only fit for indoor experiments (e.g., drag coefficient in tunnels [24]), or require a significant power supply (for e.g., drag coefficient [25] or sap flow [26]), making them unsuitable for long-term field campaigns, especially in challenging environments such as tropical forests. In this paper, we show that accelerometers are a cheap (±200 USD) and robust alternative for obtaining long-term data series of tree motion, which is used to infer tree properties and tree-related fluxes, such as mass, canopy precipitation interception, and turbulent exchange.

Accelerometers mounted on a tree trunk measure the sway movement of the tree. Tree sway is determined by tree properties such as mass, elasticity, wood density, and drag coefficient, and momentum in the atmosphere [27]. Tall, wide trees will respond differently to the same wind forcing as small, slim trees. Similarly, trees with different crown architecture (e.g., leaf size, distribution, orientation) will most likely respond differently to a similar wind load. Several studies [28,29,30,31,32,33,34,35,36] measured tree sway to determine the response to wind loads, mainly to study tree wind damage and tree failure mechanisms. Studying tree sway and bending in response to wind load is important for forestry, as storm damage to trees is a large source of economic loss [27,29]. Mayer [29] found that the primary sway of a tree (1st harmonic) is related to tree throw. Tree throw is one of the failure mechanisms that removes the whole tree, including roots, from the soil due to wind load. Mayer [29] used accelerometers to identify the peak of the 1st harmonic in the frequency spectrum and suggested that this can be changed to reduce the risk of storm damage, e.g., by cutting off tops, the crown, or chaining trees. To optimize wind shelter, and thereby reduce storm risk, Flesch and Wilson [33] used tilt sensors (which measure tree inclination) to assess the influence of silvicultural management techniques on the reduction on wind throw. By comparing frequency spectra of trees for different cutblock dimensions (clear areas in forests), they provided suggestions for maximizing risk reduction. Further development of research tools to study the origin and modes of tree failures caused by wind was done by Sellier et al. [35]. They used tilt sensors to identify peaks in the frequency spectrum of tree sway and compared different trees to study the influence of crown architecture of trees on stability.

Earlier work mainly determined frequency spectra for a single moment in time using 30 min to 1 h of data. Lohou et al. [34] presented the evolution of the acceleration frequency spectrum during a day, in order to better understand canopy–atmosphere interactions. It was shown that tree sway primarily depends on available momentum in the wind. More recent work discussed the importance of taking temporal tree dynamics into account in analyzing tree sway. Schindler et al. [36] deployed inclinometers on the trunk, and primary and secondary branches in broadleaved trees, and identified multiple vibration modes, which were linked to different parts of the tree. They discussed the importance of foliage phenology that might cause seasonal variation in the tree motion acceleration spectrum, as the tree properties determining the tree sway will change over time. Additional opportunities of using accelerometers to study temporal variations in tree sway has not been discussed until recently. Selker et al. [37] suggested that changes in the frequency spectrum were related to mass variations, resulting from leaf loss or intercepted precipitation. This was based on Stewart et al. [38], who used accelerometers for the design of hydrological measurement equipment. Stewart et al. [38] developed a rain gauge that was able to detect changes in mass due to precipitation and evaporation. By estimating the peak frequency in the wind driven motion of a bucket on a stick, precipitation amounts were estimated with an accuracy up to 1 mm. Although trees do not exhibit similar simple behavior, the general idea that mass changes influence the frequency spectrum is still valid. Llamas et al. [39] hypothesized that even diurnal variations in mass, e.g., as a result of changing in storage, might influence the frequency spectrum. Temporal dynamics in the frequency spectrum, and the analysis of other spectrum parameters in addition to the peak frequencies, are yet to be explored.

This study focuses on the analysis of tree sway time series collected for 19 trees in the Amazon rainforest over a five month period. The analysis aims to demonstrate the potential of using accelerometers to measure three crucial tree properties and responses: (1) mass variations, (2) interception of precipitation, and (3) tree–atmosphere turbulent interaction. Tree mass varies as a result of growth, leaf drop or development, and changes in water content. Tree mass changes diurnally, mainly due to variation in tree water content. During the day, transpiration exceeds water uptake during the day, leading to a decrease in water content from sunrise to sunset. At night, water uptake exceeds transpiration and water content increases again. During periods of water stress, the soil moisture availability might be insufficient for the required nocturnal refilling. In this case, the diurnal variations in tree water content, and thus mass, change as a response to water stress [17,40,41,42]. Drought causes lower transpiration and carbon assimilation rates and can eventually lead to tree mortality. Therefore, field measurements of diurnal and day-to-day mass variations of trees will increase understanding of tree response to water stress. Measurements of longer-term mass changes gives insights into tree growth and phenology. Interception of precipitation has a large impact on the hydrological cycle. Despite recent advances in simpler measurement methods (see e.g., [43]), it remains difficult to quantify interception by individual trees. This is especially challenging in tropical forests because of a substantial flow along the trunk. Finally, tree–atmosphere interaction is the driving force that determines tree sway. The momentum transfer from the atmosphere to the canopy also regulates the exchange of heat, vapor and CO${}_{2}$. Accelerometers have not explicitly been used to study temporal changes in tree–atmosphere interactions, as most measurements have been done in wind tunnels [24], or instanteanously [44].

In this paper, we discuss the concept of using accelerometers to study tree properties and responses. We give examples of several applications, which are based on measurements from a field study in the rainforest of the Brazilian Amazon. We show that measurements of tree sway can be related to tree mass variations, canopy interception of precipitation, and tree–atmosphere interaction. Finally, we provide an outlook on how accelerometers could be used in combination with auxiliary measurements to study additional tree properties and responses.

## 2. Materials and Methods

### 2.1. Theory

In previous work, it has been assumed that trees behave like damped harmonic oscillators [30,31]. To illustrate what information we can derive from tree sway measurements, we can simplify a tree as a damped mass-spring system, assuming small displacements. The displacement of such a system can be derived from Newton’s second law:

(1) $$F\left(t\right)=m\frac{d^{2}}{dt^{2}}x\left(t\right)-kx\left(t\right)-c\frac{d}{dt}x\left(t\right).$$

With external force F(t), horizontal displacement x(t), mass *m*, acceleration *a*, damping coefficient *c*, spring constant *k* and time *t*. For trees, *k* would be related to the elasticity or wood density of the tree. Higher wood density would translate in less tree sway. In the absence of external forcing *F*, the solution of a damped oscillator equation is:

(2) $$x\left(t\right)=Ae^{-\zeta 2\pi f_{0}}\sin\left(\sqrt{1-\zeta^{2}}\omega_{0}t+\phi \right).$$

With:

(3) $$\zeta =\frac{c}{4\pi \sqrt{mk}},$$

(4) $$\omega_{0}=2\pi f_{0}=\sqrt{\frac{k}{m}},$$

with amplitude *A*, natural frequency $\omega_{0}$ [rad/s] or $f_{0}$ [Hz], and phase shift ϕ. Taking the Fourier transform of Equation (1) and multiplying with the the wind force yields [38]:

(5) $$\left|H\left(\omega \right)\right|=\omega_{\alpha }\frac{1}{\sqrt{\omega_{0}^{2}-2\zeta^{2}-\omega^{2}}+4\zeta^{2}\left(\omega_{0}^{2}-\zeta^{2}\right)},$$

where H(ω) is tree motion frequency spectrum as a function of forcing frequency, and $\omega_{\alpha }$ is the amplitude of the driving wind force. It is clear from Equation (5) that tree motion, and its frequency spectrum, is a function of mass and the elasticity of the tree. Here, change in tree mass, elasticity, or a combination thereof should influence the amplitude spectrum, and the resonance frequency $\omega_{0}$ in particular.

To approach a tree as a mass-spring system is an oversimplification. In reality, trees have a significantly more complex geometry, and, therefore, can be seen as a combination of multiple, possibly nonlinear, oscillators. From the momentum balance equation, however, the spectrum of the tree response as a function of frequency $P_{y}\left(f\right)$ can be estimated using [29]:

(6) $$P_{y}\left(f\right)=H_{m}{\left(f\right)}^{2}\rho_{a}^{2}C_{D}^{2}A^{2}{\bar{u}}^{2}H_{a}{\left(f\right)}^{2}P_{u}\left(f\right)$$

with mechanical transfer function $H_{f}$, air density $\rho_{a}$, drag coefficient $C_{D}$, mean wind speed $\bar{u}$, aerodynamic transfer function $H_{a}\left(f\right)$, and power spectrum of the wind $P_{u}\left(f\right)$. In general, the aerodynamic transfer function can be approximated as $H_{a}{\left(f\right)}^{2}=1$, as there is a minimal turbulent storage term [28,29].

Stewart et al. [38] fit Equations (3) and (5) to measured spectra to determine mass and damping. However, this involves making the assumption that system behaves like a mass-spring system. While this may be valid for a simple rod with a bucket, it is not applicable in tree canopies, which tend to exhibit a broad spectrum. In this study, therefore, Equations (5) and (6) will be used to demonstrate how the frequency spectrum of the tree response depends on important tree properties, such as mass, drag coefficient, and elasticity, and to explain changes in the frequency spectra over time through variation in tree properties.

Two characteristics of the frequency spectrum can be considered: (1) the frequency peaks, and (2) the slope of the spectrum within a certain frequency range. Peaks in the frequency spectrum indicate resonance frequencies. These might be (multiples of) the natural frequencies of the tree, or of the various subsystems (branches, leaves). To identify peaks, the local maxima in the frequency spectrum are identified. For simple systems (e.g., as in Stewart et al. [38]), there is one governing peak in the spectrum that governs the signal. For higher-order systems like trees, which are more nonlinear, there is no single peak but a wide spectral response.

Therefore, we also look at the logarithmic slope [dB/Hz] of the frequency spectrum. The slope of the spectrum represents the damping of the driving wind force and can be seen as a measure of momentum transfer. As the tree movement is driven by wind, a part of the wind energy is transferred to kinetic energy in the tree. The intensity of the transfer depends on the wind speed and on the tree characteristics (such as moment of inertia, mass, and the drag coefficient). Another advantage of working in terms of slope is that it is well known that, for homogeneous isotropic turbulence, the wind energy spectrum is expected to follow the famous Kolmogorov cascade (−5/3 slope) [45].

For this research, we placed 19 accelerometers on 19 trees from August to December, 2015 in the Brazilian Amazon. We measured seven different canopy species, with two to four individuals per species (see Table 1). In this study, we present a phenomenological analysis of changes in peak frequency and the logarithmic slope. We interpret these with respect to tree properties and responses, such as tree mass, canopy interception, and canopy drag.

### 2.2. Sensor Description

**Figure 1:**
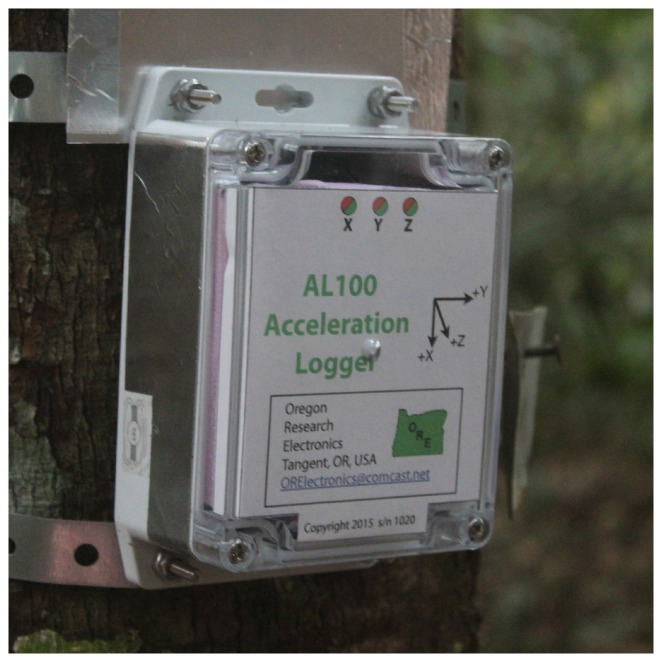
Picture of an accelerometer installed on a tree.

The accelerometer used in this study is the Acceleration Logger Model AL100 (Oregon Research Electronics, Tangent, OR, USA), which is designed to be robust and waterproof. The size is 14.5×9.2×5.5 cm, and it weighs about 400 grams (Figure 1). It can measure with a frequency of up to 25 Hz and measures the acceleration in three dimensions. A measurement frequency of 10 Hz was chosen as an optimization between accuracy and storage requirements. For further analysis, we used the acceleration component along the axis that had the largest signal, which depends on the tree species, and the orientation of the sensor, but is generally one of the horizontal axes. Depending on the sampling rate and the environment, it can log for several months on 2 C-size cell batteries. The 8 GB data card has a capacity of 320 days with 10 Hz data. Data were stored on a micro SD card, which can be easily replaced. To prevent data loss, data is written to a newly created file every day [48].

### 2.3. Measurement Setup and Protocol

**Figure 2:**
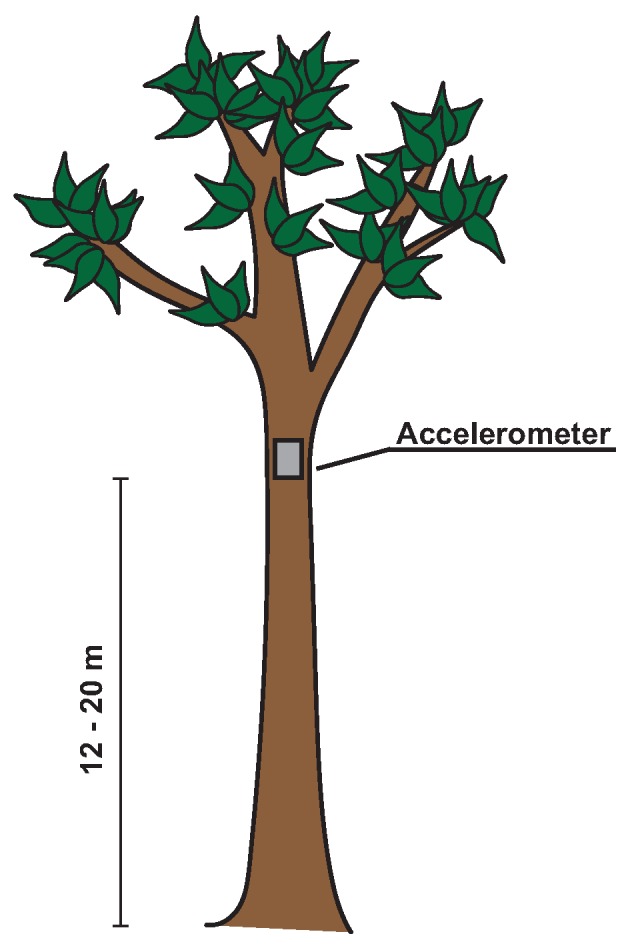
Illustration of mounting position of accelerometer in a tree.

The choice of where to place the accelerometers is govered by several factors [49]. The most important is finding a location on the tree with the largest displacement and thus the largest signal. However, tree geometry is complex, and placing the accelerometer in one of the branches does not yield a signal that is representative for the whole tree. As shown by Spatz and Theckes [50], oscillations of primary and secondary branches can significantly affect the frequency spectrum. Therefore, we placed the accelerometers on the trunk, right below the crown and the point of the main branching of the tree (see Figure 2). Other factors that influence the placing are accessibility (safety and convenience for attachment and data retrieval) and safety (against e.g., weather, and flora and fauna). For longer term measurements, it is advised to regularly read out the data and to replace the batteries.

### 2.4. Data Processing

We estimate the frequency spectrum of the horizontal, single axis acceleration using a sliding window fast Fourier transform (FFT). The spectrum was estimated every 10 min, using a window length of 30 min. To improve the frequency estimation, the raw acceleration data within the 30 min window were detrended. To prevent leakage or contamination by spectral leaking from neighboring frequencies, the data were tapered using a Hann taper on the first and last 10% datapoints of the window. We present the frequency spectra *P* in decibels:

(7) $$P=10\times log_{10}\left(\frac{p}{p_{0}}\right)$$

with spectrum *p*, and reference value $p_{0}$. For all processing, we used $p_{0}$ = 1. For this study, the slope of the frequency spectrum between 0.2 and 1 Hz was determined every 10 min. As the spectra, and thus the slope, is presented on a logarithmic scale, the slope is presented as log-log slope in Section 3.

### 2.5. Case Study Field Site and Plant Material

**Figure 3:**
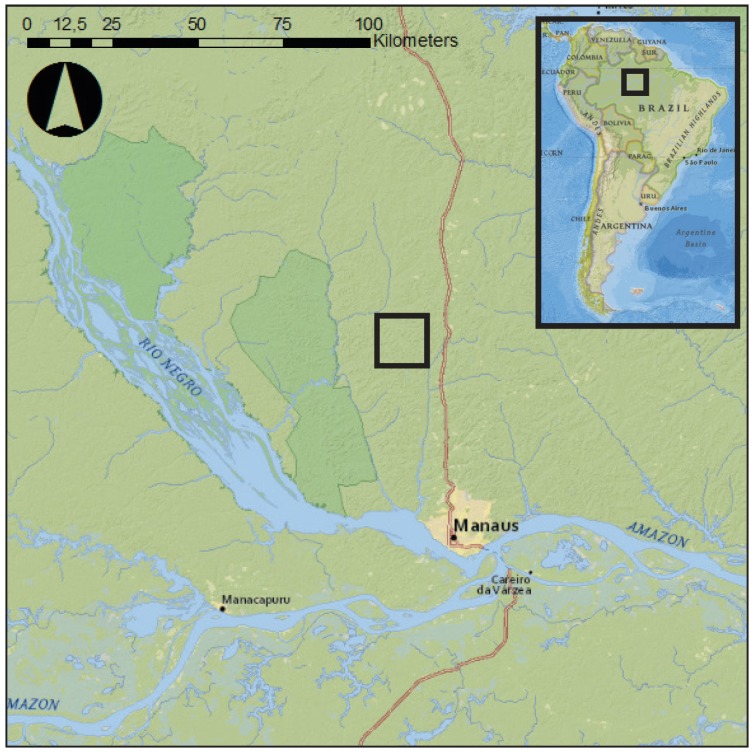
Location of the field site in the Amazon, Brazil.

This study uses data from 1 August to 31 December 2015, obtained during a field campaign at the research station in the Amazon rainforest (2.6085${}^{\circ }$ S, 60.2093${}^{\circ }$ W), 60 km Northwest of Manaus (see Figure 3). The study area is characterized by a wet tropical climate with an average dry season from June to October. During the measurement period, sunrise and sunset occurred at around 6:00 a.m. and 6:00 p.m., respectively. Additional meteorological data (wind speed, temperature, relative humidity, wind speed, precipitation) were measured every 15 min at a research tower on site. A total of 19 trees were measured with accelerometers (one per tree). Seven species were measured, with 1 to 3 individuals per species. Trees were selected to cover a broad range of heights (*h*), widths (diameter at breast height, $D_{BH}$), and wood densities ($\rho_{w}$). Wood density values were taken from the Global Wood Density Database [46,47]. Total tree height was measured using measurement tape. Tree species were determined by a classified taxonomist. Diameter at breast height (DBH) was measured using measuring tape on the day of installation of the accelerometers. Based on these dimensions, we estimated the volume (*V*) and mass (*M*) of the tree trunk using:

(8) $$V=2\pi h{\left(\frac{D_{BH}}{2}\right)}^{2},$$

(9) $$M=V\rho_{w}.$$

Note that these are approximations of the volume and mass of the tree trunk only and do not include the crown. They are intended as indicative measures of volume and mass with which we can compare and explain the results from different trees.

## 3. Results and Discussion

### 3.1. Interpretation of the Spectrum

**Figure 4:**
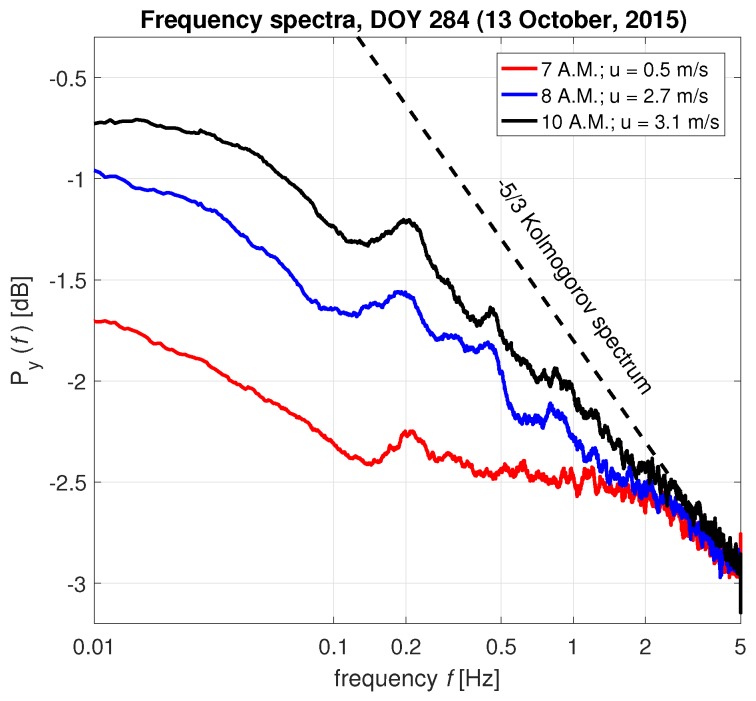
Frequency spectra of *Goupia glabra* tree no. 1 for different wind speeds on day of year (DOY) 284 (11 October 2015), including a turbulent wind spectrum (dashed black).

To illustrate how the changes in the frequency spectrum are interpreted, results are presented for two *Goupia glabra* trees. Figure 4 shows the frequency spectrum of *Goupia glabra* tree 1 at three different moments (6, 8, and 10 a.m. on 13 October 2015). For increasing wind speeds, the energy spectrum has an increasing amount of energy and several spectral peaks emerge. One of these peaks is around 0.2 Hz, which is one of the main natural frequencies of the system. The other peaks are natural frequencies of the subsystems (e.g., branches, leaves) or peaks in the forcing load. With different wind forcing, the slope of the spectrum is clearly different, which is an indicator of the degree of interaction between the wind and the tree. As the wind speed increases, the slope of the spectrum between 0.2 and 1 Hz reaches a value of almost −5/3 [dB/Hz] during higher wind speeds. This indicates that the tree sway spectrum approaches the Kolmogorov [45] wind energy spectrum characteristic of turbulent conditions. As hypothesized by Kolmogorov, turbulent motions in the inertial subrange are statistically isotropic, and the wind energy spectrum is only a function of frequency. This implies that, at high wind speeds, the tree damping is minimal compared to the forcing and could potentially be used to identify drag exerted by the tree. These results suggest that accelerometers can be used to study turbulent exchange between trees and the atmosphere.

**Figure 5:**
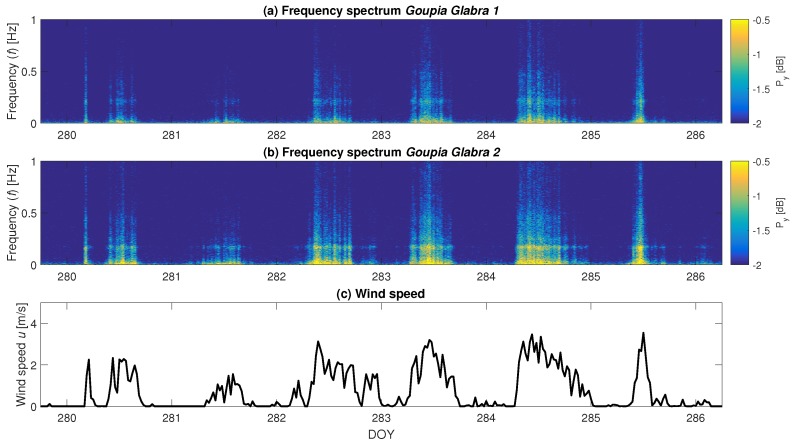
Frequency spectra of *Goupia glabra* trees nos. 1 and 2 over time from DOY 280 to 286 (9 to 15 October 2015).

Figure 5 shows the spectra of two different *Goupia glabra* trees for a five-day period, from DOY 280 to 286 (9 to 15 October 2015). Note that the frequency is plotted on a normal scale (not in log-scale as in Figure 4) At first glance, one can see that the spectra are fairly similar; however, the magnitude of the acceleration spectrum (about 0.5 dB difference) and the location of the largest frequency peak are different (0.02 Hz difference). The changes in the spectrum are mainly the result of the changing wind forcing (Figure 5c). Differences in the frequency spectra between the two trees indicate that there is variation in the amount of energy that is absorbed and damped by each tree. Given that the available wind energy is the same for both trees, the differences in acceleration spectra are due to tree specific characteristics, such as mass, catch area or drag coefficient. For the two presented *Goupia* trees, the differences between the spectra are caused by the variation in height (25 m vs. 32 m) and diameter (135 cm vs. 242.5 cm). A clear peak can be seen around 0.2 Hz for both trees, which is one of the main natural frequencies. This also illustrates the difficulties of analyzing the (changes in) natural frequency, as (1) the dynamic range of the natural frequency over time is very limited, and (2) the driving wind force needs to be higher than a certain threshold to activate this frequency.

### 3.2. Tree Mass

**Figure 6:**
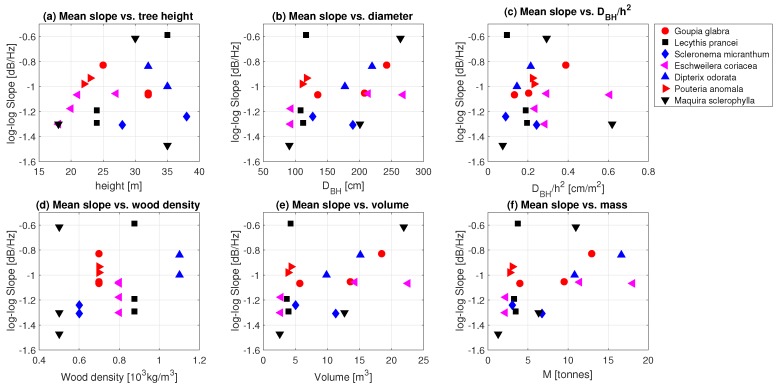
Mean slope between 0.2 and 1 Hz for each tree, plotted against (**a**) height; (**b**) diameter at breast height ($D_{BH}$); (**c**) $D_{BH}/h^{2}$; (**d**) wood density; (**e**) cylindric volume; and (**f**) cylindric mass.

The slope of the spectrum is a measure of the amount of energy transferred from the wind into tree motion. Although taller trees catch more wind, the total energy transfer also depends on other tree properties and dimensions, such as diameter, wood density, mass, and stiffness. We also look at the sensitivity to $D_{BH}/h^{2}$, as previous work has found sensitivities of the tree sway frequency spectrum to this ratio (see e.g., Moore and Maquire [27]). Figure 6 illustrates the relationships between the mean slope of the tree response frequency spectrum and tree properties. Note that we only used slopes for wind speeds higher than 2 m/s, as above this wind speed the maximum slope was reached. For the majority of the tree species, increasing height (Figure 6a), diameter at breast height $D_{BH}$ (Figure 6 b), $D_{BH}/h^{2}$ (Figure 6c), wood density (Figure 6d) volume (Figure 6e) and mass (Figure 6f) corresponds with a lower slope, indicating higher damping (and hence a higher mass or stiffness). This corresponds to the idea that taller, stiffer, more robust trees, have a higher damping of the wind load. Scleronema micranthum trees are an exception, as both height and diameter are quite different, but the mean slope is almost equal. One of the reasons for this might be the uncertainty in the estimation of the wood density. Wood densities were estimated using the Global Wood Density Database [46,47], as no measurements of trunk wood density were available. Another factor that influenced tree response is the crown structure and biomass. Schindler et al. [36], for example, discussed the differences between woodland conifers and broadleaved trees. Broadleaf trees have a more complex crown structure and their sway is therefore characterized by a less dominant main axis. Additional data on the crown architecture of the trees used in this study could therefore explain the differences in measured slope of the frequency spectrum.

In all but one species, we see a decreasing slope (increased damping) for increasing tree mass. This relationship between the slope of the spectra and the mass of the tree for each species highlights the potential to measure mass in an easy and non-destructive way. Additionally, this shows that, for some tree species, it might be possible to measure seasonal or even diurnal mass dynamics. By combining observations with modeling of the frequency spectrum, mass *m* and spring constant (tree stiffness) *k* can be determined separately. This allows the study of e.g., tree growth, tree responses to environmental stress, tree mortality, foliage development and loss, and tree water balance.

### 3.3. Effect of Precipitation

**Figure 7:**
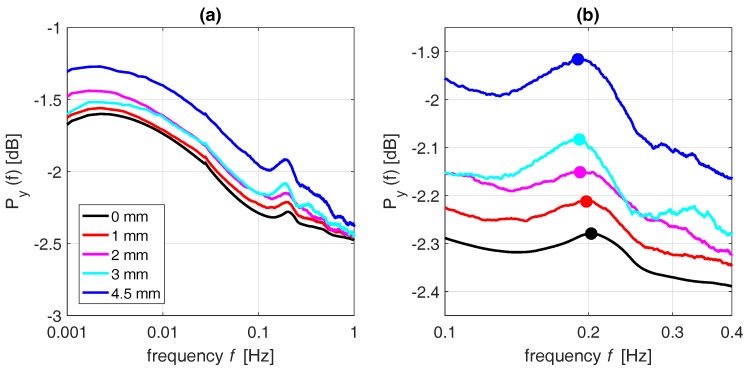
(**a**) changes in the average spectra during rain events of 0, 1, 2, 3, and 4.5 mm, and (**b**) spectra in the natural frequency range, including frequency peaks, for *Goupia glabra* tree 1.

The frequency spectrum of tree acceleration is affected by precipitation events. Precipitation is associated with increased wind speed, due to cold pools generated by e.g., rain evaporation [51], which influences the slope of the spectrum. Precipitation that is intercepted by the canopy can also lead to an increase in mass, and thus a decrease natural frequency. Figure 7a,b show the changes in the spectrum and the frequency peak for increasing precipitation amounts. Note that the spectra are average spectra for the respective precipitation amounts. Figure 7b shows the range around the frequency peak in more detail. For higher precipitation amounts, the frequency peak decreases. This is consistent with Equation (4), as the stiffness of the system does not change. A decrease in natural frequency therefore indicates a mass increase.

**Figure 8:**
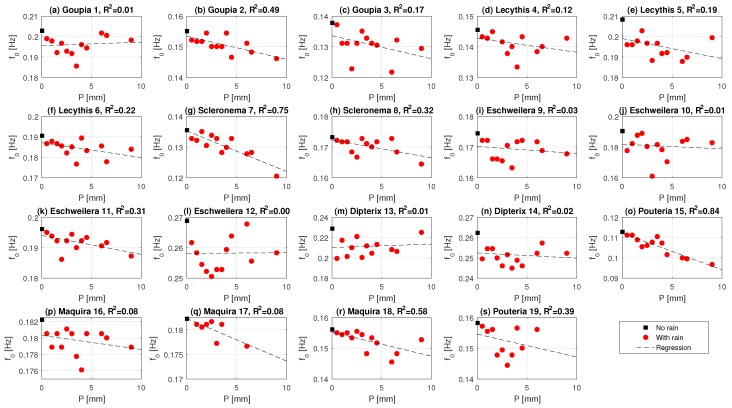
Relationship between natural frequency of the tree $f_{0}$ and the precipitation amount for all trees. Note the first point (black square) is the natural frequency without rainfall. In addition, the *y*-axis does not all have the same scale.

To further explore the sensitivity of the natural frequency $f_{0}$ to precipitation events, the difference in $f_{0}$ is plotted against the measured precipitation amount in Figure 8. It can be seen that there is some variability in the degree to which this relationship can be described with a simple linear regression. For some trees (e.g., *Goupia* 2, *Scleronema* 7, *Maquira* 16), it is clear that the natural frequency decreases linearly for higher precipitation amounts. The high sensitivity of $f_{0}$ to precipitation for the *Scleronema* and *Maquira* trees might also be explained by additional mass increase through absorption of water. Both species are light wooded species, and, during precipitation events, these species refill internal storage (parenchyma tissue) through root water uptake. This is not the case for all trees. Some trees seem to have a change in the relationship between $f_{0}$ and precipitation amount. For example, for *Goupia* 1, *Eschweilera* 12, *Dipterix* 14, and *Pouteria* 19, $f_{0}$ seems to be linearly related to precipitation between 0 and 3–4 mm. For higher precipitation amounts, the relationship becomes very uncertain.

**Figure 9:**
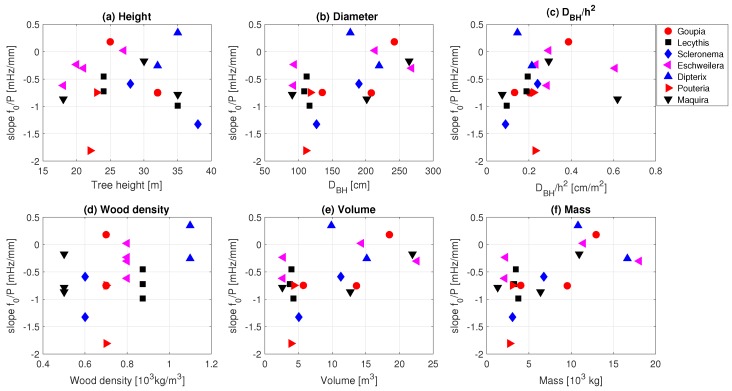
Slope of the change in natural frequency $f_{0}$ with precipitation amount [mHz/mm] against (**a**) tree height; (**b**) diameter at breast height; $h^{2}/D_{BH}$; (**d**) wood density; (**e**) volume; and (**f**) mass.

Variations in the sensitivity of $f_{0}$ to precipitation might be explained by differences in tree height, crown architecture, or other tree properties. Figure 9 therefore presents the slope of the linear regressions (change in $f_{0}$ as a function of precipitation, see Figure 8) for each tree. To study what determines the linearity of this relationship, the slope is shown as a function of height, diameter at breast height, $h^{2}/D_{BH}$, wood density, volume and mass. A steeper slope indicates a higher sensitivity to precipitation.

From Figure 9, it can be seen that the sensitivity of a tree to precipitation amount is influenced by both the mechanical properties of the trees and the location in the canopy. In Figure 9a, it can be seen that the largest trees have the highest sensitivity (−0.5 to −1.5 mHz/mm for trees higher than 30 m), which can be explained by the larger amount of precipitation intercepted by the upper canopy layers. Trees with larger diameters have a lower sensitivity (Figure 9b). This is expected, as the slope is proportional to moment of inertia, which varies with the 4th power of the diameter. Trees with larger wood density and mass (Figure 9d,f) all have a lower sensitivity to increased precipitation. Wood density is positively related to the elasticity of the tree [52], and one would therefore expect a lower sensitivity to precipitation for trees with higher wood density. In addition to the elasticity, the moment of inertia considerably affects tree displacement. This is largely influenced by the tree diameter, which explains why wider trees are less sensitive to precipitation. Finally, the shorter trees intercept less precipitation than those above them.

The weaker dependence of $f_{0}$ on precipitation amount at higher precipitation values (>2–4 mm) could be related to precipitation intensity. Higher intensity rainfall results in splashing, causing drops to fall off of the leaves. Canopy architecture also has a significant influence on the sensitivity to rainfall. Intercepted rainfall depends on the location, orientation, and size of the leaves. The sensitivity of the tree sway spectrum to intercepted rainfall is in turn influenced by leaf orientation, shape, and position with respect to the trunk. Additional information on canopy architecture can give insight into its influence on sensitivity of the $f_{0}$ to intercepted rainfall. This preliminary analysis suggests that it might be able to estimate canopy interception by analyzing the change in the frequency spectrum.

### 3.4. Energy Transfer from Wind to Tree Sway

Energy transfer between the atmosphere and vegetation has a significant influence on biotic and abiotic processes, such as tree mortality, and exchange of water, heat and CO${}_{2}$ [53]. The amount of energy that is transferred from wind to tree motion is significantly influenced by the drag coefficient of a tree. The drag coefficient is in turn a function of tree architecture, geometry, and dimensions [29,36]. As we show in Section 1, the slope of the frequency spectrum is a measure of energy transfer from the wind to the tree. As current field methods for measuring canopy drag [25] are difficult to apply for extended periods, especially in remote and challenging locations, we can use the slope as an approximation and analyze its variations with wind speed, dynamics in time, and differences between trees.

**Figure 10:**
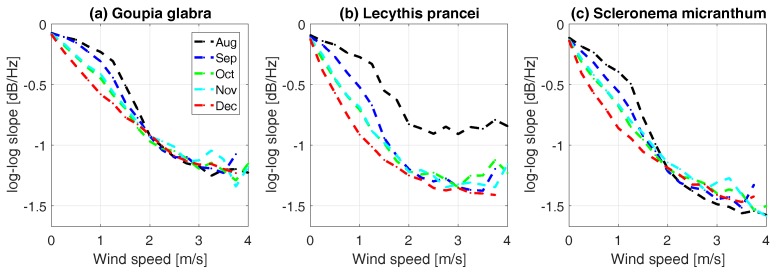
Average slope as function of wind speed from August to December, 2015, for (**a**) *Goupia glabra*; (**b**) *Lecythis prancei*; and (**c**) *Scleronema micranthum*.

Figure 10 presents the monthly averaged slope from August to December, 2015, for a *Goupia glabra* (a), *Lecythis prancei* (b), and *Scleronema micranthum* (c) trees. Drag coefficient is a function of wind speed [54], as vegetation streamlines and reconfigures as wind speed changes [25,44,55]. Generally, higher wind speeds result in lower drag coefficients for trees. As wind speed increases from 0 to 1.5 m/s, the slope, and thus energy transfer, increases. This means that, for increasing available wind energy, relatively less energy is transferred into tree motion, indicating a lower drag coefficient. The rate of energy transfer clearly varies per month as function of wind speed and between trees. For all three trees, the energy transfer seems to increase between August and December, 2015. This is most likely a reflection of phenological changes, such as leaf flushing or drop [56]. In addition, the differences between trees are considerable. The inflection point for the *Scleronema micranthum* tree is less clear, indicating a higher drag coefficient than the *Goupia glabra* and *Lecythis prancei* trees. The *Lecythis prancei* shows the largest dynamic range over time, with a difference in maximum slope of 0.6 for wind speeds over 2 m/s between August and December 2015, against an almost negligible difference for the *Goupia glabra* tree. Recall from Equations (1)–(6) that drag coefficient, catch area and mass are important parameters that determine the energy transfer from atmosphere to tree. The large variations observed for *Lecythis* are therefore likely to be caused by changing tree properties—for example, leaf drop between August and September, 2015.

We show that accelerometers might be a useful method to study the interaction of a tree with the atmosphere. In large eddy simulations and meteorological models, the drag coefficient is often assumed constant in time and space [53,57,58]. Results presented here suggest that tree drag coefficient is variable in space and time, following changes in tree leaf biomass changes. Combining our results with auxiliary data, particularly high temporal resolution wind data, may allow us to derive the absolute drag coefficient for each tree. In addition, additional information on canopy architecture will give insight into the influence of canopy architecture on the relationship between the slope and wind speed, and, therefore, on energy transfer from atmosphere to individual trees. Future work will focus on further analysis on the temporal, spatial, and between-tree variation in energy transfer between atmosphere and trees.

### 3.5. Synthesis and Outlook

We show several examples of information that can be extracted from analyzing frequency spectra of tree motion. We demonstrate that we can detect differences in mass and turbulent exchange between and within species, through the relationships between the slope of frequency spectra (damping) and mass. More interestingly, this might allow analyzing seasonal or even diurnal variation in tree mass. This means that accelerometers might be used to track growth of trees or monitor changes in diurnal mass affiliated with changing water content. Potentially, accelerometers can give more insights into the changes in tree properties and dynamics in response to drought.

Further exploration of the possibilities of quantifying canopy interception of precipitation might contribute to a better understanding of the water cycle in forest ecosystems. Interception is one of the most important parts of the hydrological cycle. Unfortunately, it is also difficult to measure, especially on larger scales. Validation studies, including additional throughfall, stem compression measurements [43], above and below canopy evaporation measurements [59], or stable isotopes to separate evaporation fluxes [60], will shed light on the possibilities of measuring canopy interception of individual trees using accelerometers. Note that rainfall events that occur during low wind speeds will remain difficult to detect, due to a lack of tree sway.

A novel approach is presented, using accelerometers to study the interaction between trees and the atmosphere. So far, most studies derived drag coefficients from wind speed and eddy flux measurements. Additional long time series of tree sway will increase understanding of energy and momentum transfer by individual trees. Dynamics in the energy absorption and damping might in future applications also be coupled to seasonal and diurnal mass variations due to growth, or water stress—for example, to study the changes in canopy–atmosphere interaction during the dry season.

Other applications might include studying tree mortality, and wind-damage. As accelerometers are cheap and robust sensors, they might be used to study tree dynamics and properties for validation of remote sensing products in more challenging environments.

## 4. Conclusions

Accelerometers were deployed on a broad range of trees in the Amazon. They are simple, cheap and robust sensors that can last in challenging environments. We show how they can be applied in a tropical forest, and, how with simple data processing algorithms, valuable information on tree properties and responses can be extracted.

The slope of the frequency spectrum is related to tree mass. For all but one species, increasing tree mass clearly resulted in a lower slope. Future work will use additional data, such as throughfall, stem compression, evaporation and transpiration, to relate the frequency spectrum to mass changes due to growth on seasonal, and changing water content on a diurnal scale.

The frequency spectrum shows a clear response to precipitation events. The observed decrease in natural frequency is associated with an increased mass, due to water stored on the canopy. For several trees, the relationship between natural frequency and precipitation amount is linear, which highlights the potential of using accelerometers to estimate canopy interception by individual trees.

Preliminary results demonstrated variability in canopy–atmosphere interaction in space and time. Changes in the frequency spectrum can be associated with variations in tree drag coefficient. The results suggest that the tree drag coefficient is more variable than currently assumed.

The affordable, easy-to-apply, robust accelerometers used in this study provide a promising complementary technique to current measurement techniques. The results presented here demonstrate that low-cost accelerometers can yield valuable insight into tree properties and responses. Longer deployments and their combination with other instruments in experimental studies is encouraged to reveal their full potential and to identify additional applications in vegetation monitoring.

## Figures and Tables

**Table 1:** Tree characteristics: tree number, scientific name, wood density [46,47], estimated total height and diameter at breast height (DHB).

Tree No.	Name	[10${}^{3}$ kg/m${}^{3}$]	Wood Density High–Low	Height [m]	$D_{\mathrm{BH}}$ [cm]
1–3	*Goupia glabra* (brevi-deciduous)	0.7	Low	25–32	135.0–242.5
4–6	*Lecythis prancei* (evergreen)	0.875	Intermediate	24–35	108.4–116.5
7–8	*Scleronema micranthum* (evergreen)	0.6	Low	26–38	81.0–189.5
9–12	*Eschweilera coriacea* (evergreen)	0.8	Intermediate	18–27	92.4–268.0
13–14	*Dipterix odorata* (evergreen)	1.1	High	32–35	177.0–219.5
15–16	*Pouteria anomala* (evergreen)	0.7	Low	22–23	111.0–117.5
17–19	*Maquira sclerophylla* (evergreen)	0.5	Low	18–35	90.6–264.0
